# Genomic prediction and GWAS of yield, quality and disease-related traits in spring barley and winter wheat

**DOI:** 10.1038/s41598-020-60203-2

**Published:** 2020-02-25

**Authors:** Hsin-Yuan Tsai, Luc L. Janss, Jeppe R. Andersen, Jihad Orabi, Jens D. Jensen, Ahmed Jahoor, Just Jensen

**Affiliations:** 1 Department of Marine Biotechnology and Resources, National Sun Yat-Sen University, Kaohsiung City, Taiwan; 2 Center for Quantitative Genetics and Genomics, Department of Molecular Biology and Genetics, Aarhus University, Tjele, Denmark; 3 Nordic Seed, Galten, Denmark; 4 Department of Plant Breeding, Swedish University of Agricultural Sciences, Alnarp, Sweden

**Keywords:** Plant sciences, Plant breeding

## Abstract

Genome-wide association study (GWAS) and genomic prediction (GP) are extensively employed to accelerate genetic gain and identify QTL in plant breeding. In this study, 1,317 spring barley and 1,325 winter wheat breeding lines from a commercial breeding program were genotyped with the Illumina 9 K barley or 15 K wheat SNP-chip, and phenotyped in multiple years and locations. For GWAS, in spring barley, a QTL on chr. 4H associated with powdery mildew and ramularia resistance were found. There were several SNPs on chr. 4H showing genome-wide significance with yield traits. In winter wheat, GWAS identified two SNPs on chr. 6A, and one SNP on chr. 1B, significantly associated with quality trait moisture, as well as one SNP located on chr. 5B associated with starch content in the seeds. The significant SNPs identified by multiple trait GWAS were generally the same as those found in single trait GWAS. GWAS including genotype-location information in the model identified significant SNPs in each tested location, which were not found previously when including all locations in the GWAS. For GP, in spring barley, GP using the Bayesian Power Lasso model had higher accuracy than ridge regression BLUP in powdery mildew and yield traits, whereas the prediction accuracies were similar using Bayesian Power Lasso model and rrBLUP for yield traits in winter wheat.

## Introduction

Both wheat (*Triticum aestivum L*.) and barley (*Hordeum vulgare L*.) are major cereal crops worldwide, ranking as the second and fourth in total grain production. The global wheat and barley production amounted to 736 and 146 million metric tons in 2016, with 21% and 41% contributed by European countries, respectiely^1^. Due to their economic role worldwide, the advancement of economically important traits of wheat and barley is indispensable, in order to meet the food demands of growing human population.

Recent developments in genome sequencing technologies allow plant breeders to characterize the genetic architecture of economically important cereal crops. The reference genomes are now available in both spring barley and winter wheat^2–4^, raising the possibilities for plant breeders to introduce genome-assisted selection to both crops. Several high-density SNP microarrays enabling association mapping, and increase the resolution of quantitative trait loci (QTLs) mapping using hundreds of thousands of genetic polymorphisms throughout the entire genome, are now available. For example, there are several SNP arrays with the varying number of genetic variants customized for both barley and wheat^5–9^.

Traditionally, the molecular-assisted breeding, using molecular biology techniques, such as gene identification and gene functional characterization, to explain the genetic mechanism of economically important traits has been used in crops. However, several studies evidenced that most traits of commercial interest in wheat and barley are highly polygenic, with many QTLs that each only account for a small proportion of total genetic variances^10,11^. This severely limits the application of traditional molecular-assisted selection in cereal crops.

Due to the SNP microarray becoming commercially available, and the genotyping cost is continually decreasing, the genome-assisted breeding has become an applicable method to advance the efficiency of selection. Genes or QTLs with large effects can be mapped using linkage mapping or genome-wide association studies (GWAS), which are mainly based on LD between the genetic marker and causal mutation. Compared with linkage mapping, GWAS can account for higher allelic diversity at the corresponding loci, and can exploit ancestral recombination events in a population or species^12,13^. Therefore, GWAS has become a better choice to identify the genetic variants or QTLs associated with phenotypes of interest. By using GWAS, numerous putative genetic regions associated with important economic traits and diseases have been documented in plants^10,11,13–15^. However, GWAS methods are usually implemented using single-trait-based studies, the multivariate trait records are compressed to single score (such as selection index), which may limit the detection of pleiotropic genetic variants across traits^16^. Besides, genotype by environment (GxE) effects, such as the interaction between genotype and location, usually have considerably influenced grain yield performance in field experiments. Several studies included GxE effects to characterize the GxE, and to increase the prediction accuracy of genomic prediction in plant breeding. However, relatively few studies characterized the effect between genotypes and locations using GWAS model to evaluate the SNP effects in different locations^17,18^.

The genomic-assisted breeding, such as genomic selection (GS), has been extensively implemented in animal and plant breeding^19–21^. The concept of GS is to utilize a large set of, usually anonymous markers, spread over the whole genome such that every QTL are in linkage disequilibrium (LD) with at least one marker. By accumulating effects of markers or haplotypes, this approach allows plant breeders to assess the genomic estimated breeding values (GEBVs) of breeding lines at a stage in plant breeding programs using early generations of the plant breeding program. This is especially advantageous for traits that cannot be assessed on single or few plants, and for traits that are difficult to measure. Several reviews on GS in plant breeding has been reported^21–23^. However, how to advance the accuracy of GEBVs prediction to select the elite breeding lines is still an important topic for plant breeders.

Several statistical approaches have been proposed for efficient prediction of genomic breeding values in genomic-assisted breeding programs. For instance, ridge regression BLUP model (rrBLUP), also called as SNP-BLUP model, is a commonly employed model in genomic evaluation^24^. For rrBLUP, the GEBVs of breeding lines can be estimated as the sum of all predicted additive marker effects. The marker effects are usually assumed to have a normal distribution with the same variance for all marker effects. The marker effects are equally shrunken towards to a small number, which closes to but not zero, to fit with the total genetic variance, and to avoid over-fit in the model when including large amounts of genetic markers in the analysis. This assumption may lead to markers with large effect can be shrunken too much, but small marker effects are not shrunken enough. A Bayesian model provides an alternative way to adjust the prior distribution of marker effects. Rather than setting as normal distribution as the rrBLUP model, the Bayesian models can assign a distribution so that markers with large effects are shrunken less, and small marker effects are shrunken more. As a result, compared with Bayesian model, rrBLUP is likely not the ideal method to evaluate marker effects when some markers have large effects while most of them have small effects. However, drawbacks of Bayesian model can be very tedious computations, and in some cases the prediction accuracy is not significantly more accurate than predictions using rrBLUP, when the genomic relationships between individuals in the training population are high, or the genomic relationship between training population and validation population is close^24,25^.

The aims of this study were: (1) to identify genetic loci associated with grain yield traits, quality traits, and disease-resistance traits by using single trait and multiple trait GWAS; (2) to include the genotype-by-location information in the GWAS model to evaluate the marker effects toward grain yield performance in winter wheat tested in three different experimental locations and (3) to develop models for genomic prediction of traits of interest using rrBLUP and Bayesian Power Lasso model.

## Results

### General descriptive statistics

**Figure 1:**
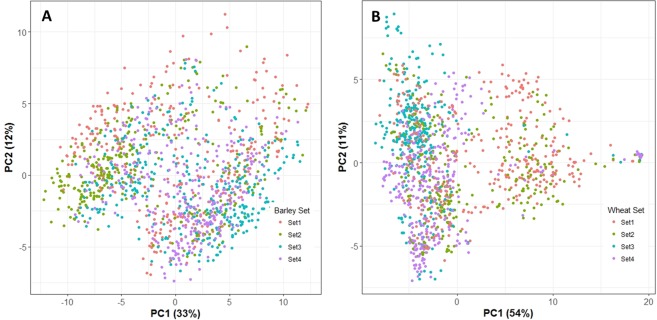
The PCA plot for (**A**) spring barley, and (**B**) winter wheat. The PCA plot based on genomic relationship matrices (GRM) showed that all breeding lines across 4 sets were almost mix together without clear clusters.

**Figure 2:**
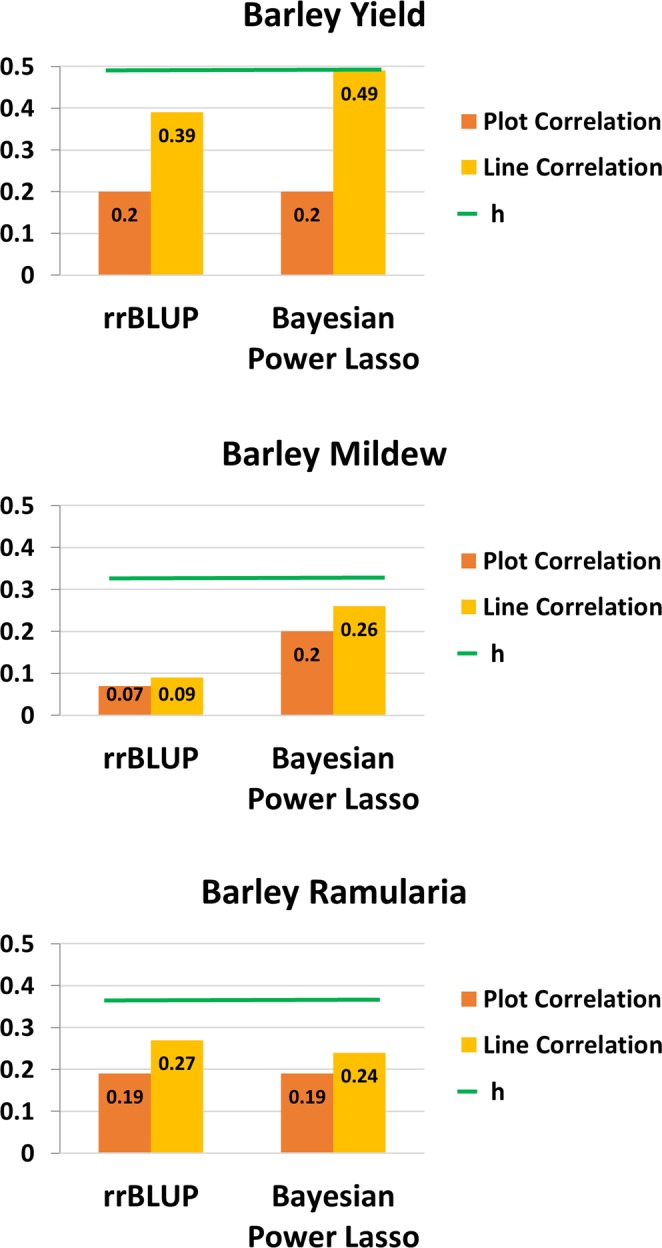
Genomic prediction model comparison of traits in spring barley breeding lines. The plot correlation (ρ(**y**_**c**_, **ĝ**)) was estimated by calculating the correlation between the plot records corrected for the fixed effect (**y**_**c**_), and genomic predicted breeding values (**ĝ**). The line correlation (ρ(${\bar{{\bf{y}}}}_{{\bf{c}}}$, **ĝ**)) was estimated by calculating the correlation between the average of plot records corrected for the fixed effect based on the same line (${\bar{{\bf{y}}}}_{{\bf{c}}}$), and genomic predicted breeding values (**ĝ**). Maximum correlation, h, represents the square root of the narrow-sense plot genomic heritability, and is shown as green bar over the correlation.

**Figure 3:**
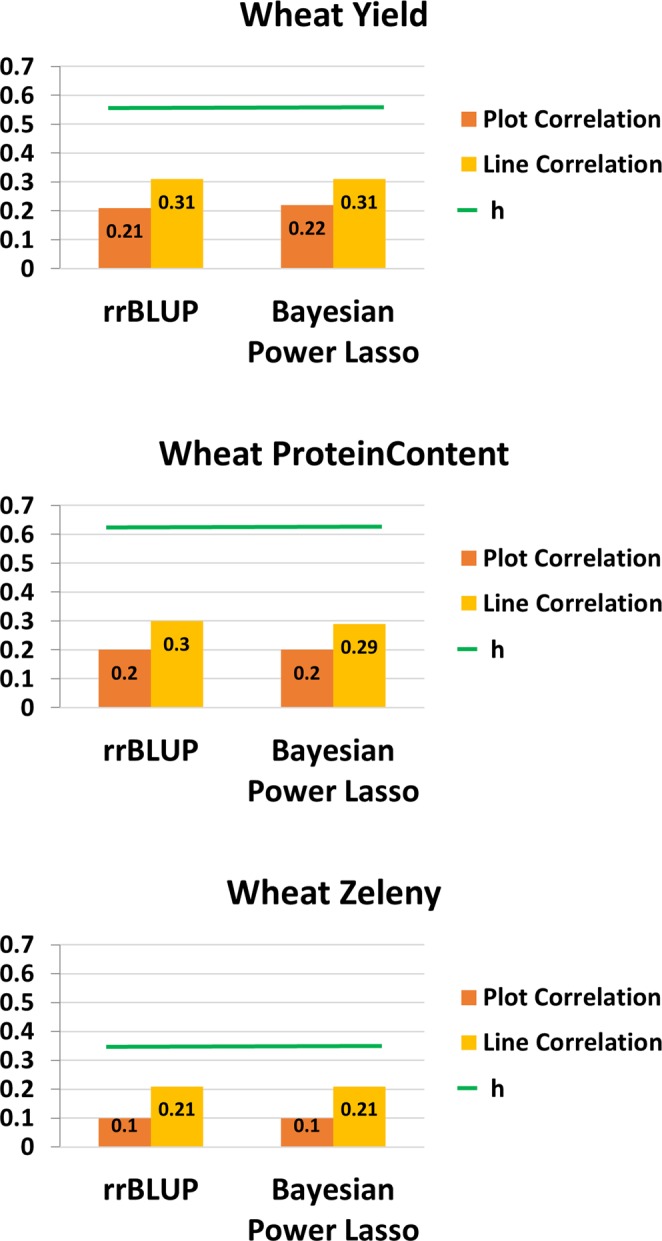
Genomic prediction model comparison of traits in winter wheat breeding lines. The same denotations are given in Fig. 5.

General descriptive statistics of the traits analyzed are detailed in Table 1. There were five traits studied in spring barley, and five traits in winter wheat. The number of plots in powdery mildew and ramularia were less than for other traits because only one plot was recorded per year and per location in this study. In general, the distribution of all traits showed close to normal distributions, but powdery mildew was skewed as 90% of all records were scored as no infection. The PCA pointed out that the first two principal components explained 33% and 12% of the total variance among markers for spring barley, and 54% and 11% for winter wheat (Fig. 1). Based on the genomic information, the results showed that lines, in general, were grouped in families in both spring barley and winter wheat. The heat-map of the genomic relationship (data not shown as similar results were reported^26,27^) using similar dataset also lead to the same result for both crops^26,27^. The square root of narrow-sense plot genomic heritabilities for all traits are given in Fig. 2 for barley and in Fig. 3 for winter wheat. For spring barley, the narrow-sense plot genomic heritability was 24% in yield, 11% in powdery mildew, and 13% in ramularia. For winter wheat, the narrow-sense plot genomic heritability was 33% in yield, 39% in protein content, and 12% in Zeleny value. The phenotypic correlation of traits were generally lower than genetic correlation across different traits in both species (Tables 2 and 3).

**Table 1:** Descriptive statistics of spring barley and winter wheat phenotypic records. The units of each trait are given in phenotype collections in materials and methods.

Species	Trait*	No. of Plots	Mean (SD)	Min.	Max.
**Barley**	Yield	15376	6.60 (0.8)	4.2	9.4
	Straw breaking	9102	3.60 (2.03)	1	9
	Powdery Mildew	2995	1.09 (0.6)	1	7
	Ramularia	2703	3.91 (1.0)	1	7
	Lodging	8489	2.98 (1.87)	1	9
**Wheat**	Yield	13329	8.62 (0.9)	3.9	14.8
	Moisture	13300	15.96 (1.68)	11.9	22.9
	Protein content	13300	9.44 (0.9)	6.7	15.1
	Zeleny value	8036	23.12 (6.6)	5	54
	Starch Content	9479	68.79 (1.92)	60	77

**Table 2:** (**a**) The genetic correlation (underlined) and phenotypic correlation (*italic*) between the disease-related traits in spring barley. (**b**) The genetic correlation (underlined) and phenotypic correlation (*italic*) between the yield-related traits in spring barley.

(a) Barley	Powdery mildew		Ramularia
Powdery mildew	—		*0.05*
Ramularia	0.29		—
**(b) Barley**	**Yield**	**Lodging**	**Straw breaking**
Yield	—	*0.19*	*0.06*
Lodging	0.4	—	*0.34*
Straw breaking	0.06	0.71	—

**Table 3:** (**a**) The genetic correlation (underlined) and phenotypic correlation (*italic*) between the quality-related traits in winter wheat. (**b)** The genetic correlation (underlined) and phenotypic correlation (*italic*) between the yield-related traits in winter wheat.

(a) Wheat	Protein Content		Zeleny value
Protein Content	—		*0.28*
Zeleny value	0.84		—
**(b) Wheat**	**Yield**	**Moisture**	**Starch Content**
Yield	—	*0.12*	*0.03*
Moisture	0.55	—	*0.32*
Starch Content	0.53	0.21	—

### GWAS to Identify SNPs associated with traits in spring barley and winter wheat

**Figure 4:**
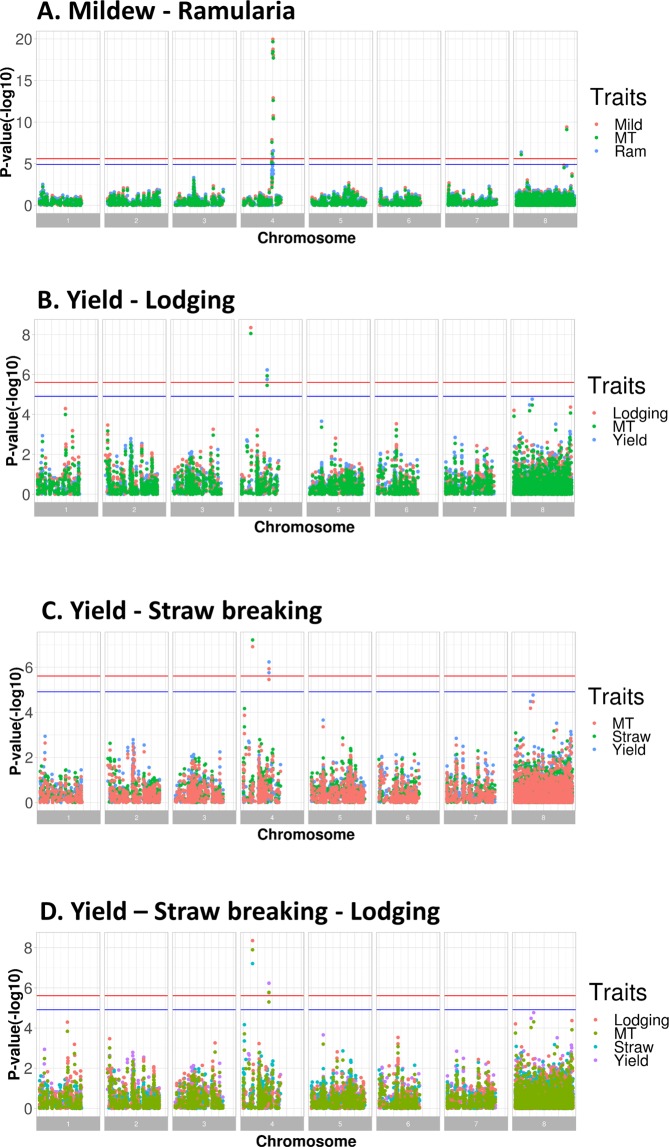
Manhattan plot of univariate and multivariate GWAS in spring barley breeding lines. The X-axis is the chromosome position, and chromosome 8 is unmapped SNPs. The Y-axis is P-value (−log_10_). The blue line is the significance threshold at 5%, and the red line is the significance threshold at 1%. MT represents multivariate GWAS result. The legend using the trait name is univariate GWAS result.

**Figure 5:**
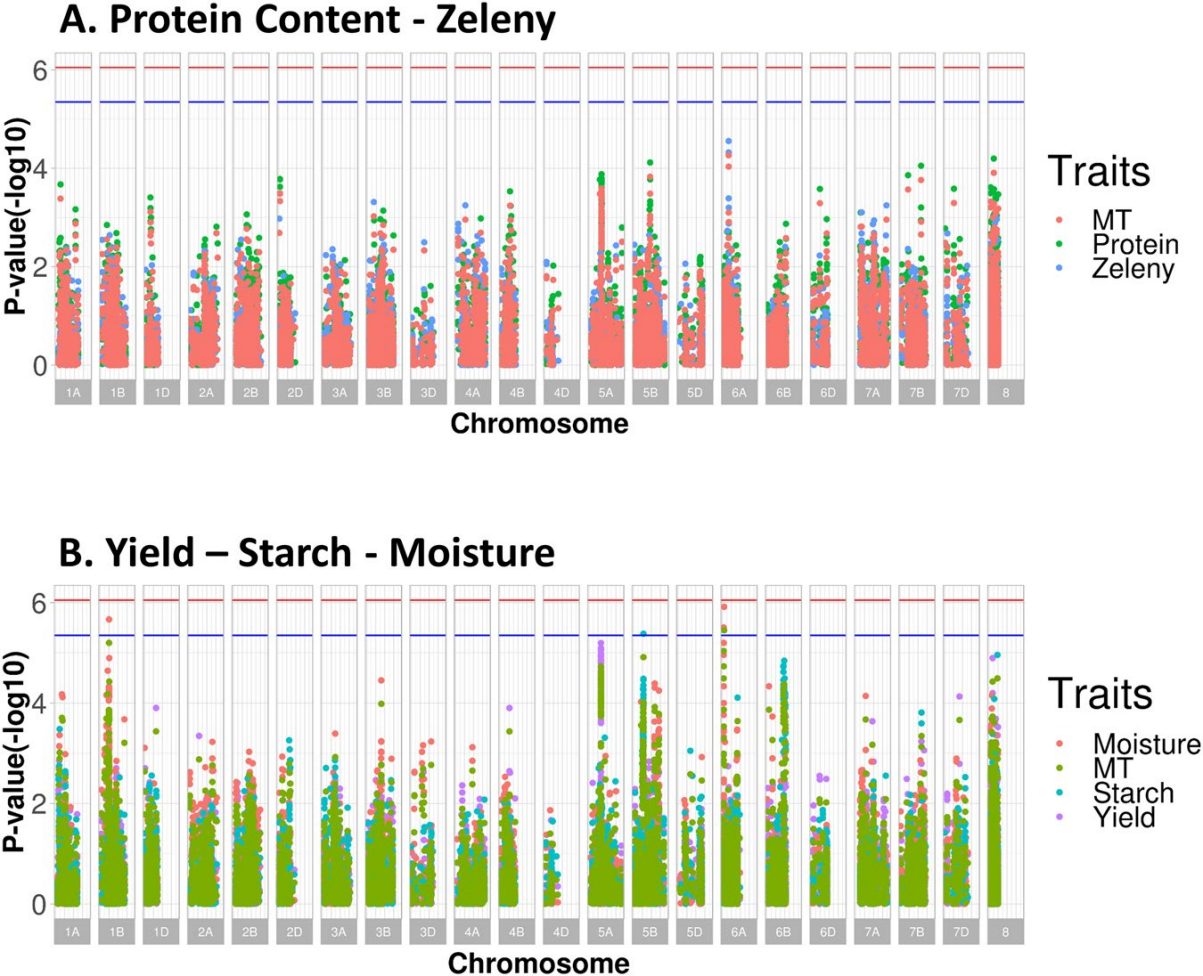
Manhattan plot of univariate and multivariate GWAS in winter wheat breeding lines. The blue line is the significance threshold at 5%, and the red line is the significance threshold at 1%. MT represents multivariate GWAS result. The legend using the trait name is univariate GWAS result. Chromosome 8 represents positional unknown SNPs.

GWAS were performed using single marker regression to estimate SNP effects for each trait. The family structure was taken into account in the model using the genomic relationship matrix based on all validated genetic markers in both species, respectively. The Manhattan plots of univariate GWAS (or named as single trait GWAS) and multivariate GWAS (or named as multiple trait GWAS) for tested traits are given in Fig. 4 for spring barley, and in Fig. 5 for winter wheat. The lists of all genome-wide significant markers are given in Table 4 for univariate GWAS, and Table 5 for multivariate GWAS. The genome position unknown SNPs were represented by chromosome 8, as depicted in the legend in Figs. 4 and 5.

**Table 4:** Genome-wide significant markers in univariate GWAS in spring barley and winter wheat (P < 0.05).

	Trait	Marker	Chromosome	Position(cM)	P-value	Genomic Effect*
**Barley**	Powdery Mildew	SNP1	4 H	100.68	1.09E-20	10.70
		SNP2	4 H	101.28	1.72E-19	10.30
		SNP3	4 H	99.68	2.73E-19	10.00
		SNP4	4 H	102.38	9.75E-19	10.20
		SNP5	4 H	101.68	1.23E-13	6.70
		SNP6	4 H	102.08	1.68E-11	5.55
		SNP7	4 H	102.18	1.95E-11	5.53
		SNP8	Unknown	Unknown	3.89E-10	5.03
	Ramularia	SNP6	4 H	102.08	2.69E-07	7.03
		SNP7	4 H	102.18	2.92E-07	7.00
		SNP9	Unknown	Unknown	4.01E-07	6.11
	Yield	SNP10	4 H	88.84	5.85E-07	0.75
		SNP11	4 H	88.84	5.85E-07	0.75
		SNP12	4 H	88.84	5.85E-07	0.75
		SNP13	4 H	88.64	1.76E-06	0.68
	Starch	SNP14	4 H	32.24	6.19E-08	18.60
	Lodging	SNP14	4 H	32.24	4.44E-09	12.70
**Wheat**	Moisture	SNP15	6 A	40.8	1.22E-06	1.34
		SNP16	1B	201.25	2.16E-06	1.44
		SNP17	6 A	30.04	3.13E-06	1.23
	Starch Content	SNP18	5B	222.57	4.17E-06	4.09

*:The genomic effect value is multiplied by 10^3^.

**Table 5:** Genome-wide significant markers in multivariate GWAS in spring barley and winter wheat (P < 0.05).

	Trait Combination	Marker	Chromosome	Position(cM)	P-value
**Barley**	MildewRamularia	SNP1	4 H	100.68	2.18E-20
		SNP2	4 H	101.28	3.44E-19
		SNP3	4 H	99.68	5.46E-19
		SNP4	4 H	102.38	1.95E-18
		SNP5	4 H	101.68	2.45E-13
		SNP6	4 H	102.08	3.36E-11
		SNP7	4 H	102.18	3.91E-11
		SNP8	Unknown	Unknown	7.78E-10
		SNP19	4 H	97.38	2.59E-08
		SNP20	4 H	98.65	6.51E-07
		SNP9	Unknown	Unknown	8.01E-07
		SNP21	4 H	97.29	6.64E-06
		SNP22	4 H	97.19	6.70E-06
		SNP23	4 H	97.19	9.18E-06
	YieldStarchLodging	SNP14	4 H	32.24	1.27E-08
		SNP10	4 H	88.84	1.68E-06
		SNP11	4 H	88.84	1.68E-06
		SNP12	4 H	88.84	1.68E-06
		SNP13	4 H	88.64	5.04E-06
**Wheat**	MoistureStarchYield	SNP15	6 A	40.8	3.57E-06

*The markers were not identified in univariate GWAS (*cf*. Table 4).

In general, the Q-Q plot for each analysis showed that the observed −log_10_(P-value) was close to expected −log_10_(P-value), but in the tail of the distribution dots were deviated from observed value in most cases indicating that significant marker effects were found. Also, for traits, such as moisture and protein content in winter wheat, p-values were slightly inflated (See Q-Q plots in Supplementary Information).

#### Spring barley

For barley, most genome-wide significant markers were mapped on chromosome 4H, and some significant markers were at unknown genome position. For powdery mildew, seven markers on chromosome 4H and one unmapped marker passed the genome-wide significance threshold (P < 0.05), explaining 8.9% of the total genetic variance. For ramularia, there were two markers on chromosome 4H and one with unknown position showed genome-wide significance, explaining only 0.9% of the total genetic variance. The multiple trait GWAS jointly analyzing powdery mildew and ramularia showed that the results were consistent with the univariate GWAS, as the same significant markers were identified on chromosome 4H and one marker with unknown position (Fig. 4). For yield, four markers located on chromosome 4H passed the genome-wide significance threshold, explaining 1% of the total genetic variance. For straw breaking and lodging, the same marker (marker name: SNP14) was significantly associated with the QTL on chromosome 4H across two traits. Overall, the multivariate GWAS using P-value and phenotypic correlation information from two traits (Figs. 4B,C) and three traits (Fig. 4D), showed that the identified significant markers were similar to univariate GWAS across different analyses.

#### Winter wheat

For protein content and Zeleny value in winter wheat, the most significant SNPs were found on chromosome 6A, but no genome-wide significant SNPs were associated with any of the traits. This was also observed from the multivariate GWAS results (Fig. 5). For moisture, there were two markers on chromosome 6A and one marker on chromosome 1B passing the genome-wide significance threshold (P < 0.05), explaining 0.5% of total genetic variance (Table 4, moisture section). For starch content, a genome-wide significant marker was found on chromosome 5B (Table 4, starch content section), and notably, there were several markers located on chromosome 6B (nine SNPs), 5B (three SNPs), and unmapped region (one SNP), nearing but not passing the genome-wide significance threshold (P < 0.05), explaining 2.2% of the total genetic variances (SNP markers not detailed in Table 4 as they are not passing the genome-wide significance threshold). For yield, no genome-wide significant markers were found, however, there were six markers on chromosome 5A closing to the significance threshold (P < 0.05), accounting for 1% of total genetic variance. There was only one marker showing genome-wide significance on chromosome 6A using multivariate GWAS, based on the joint multivariate analysis of moisture, starch, and yield (Fig. 5B).

### GWAS model including genotype-location information to evaluate SNP effect in three different locations in yield in winter wheat

**Figure 6:**
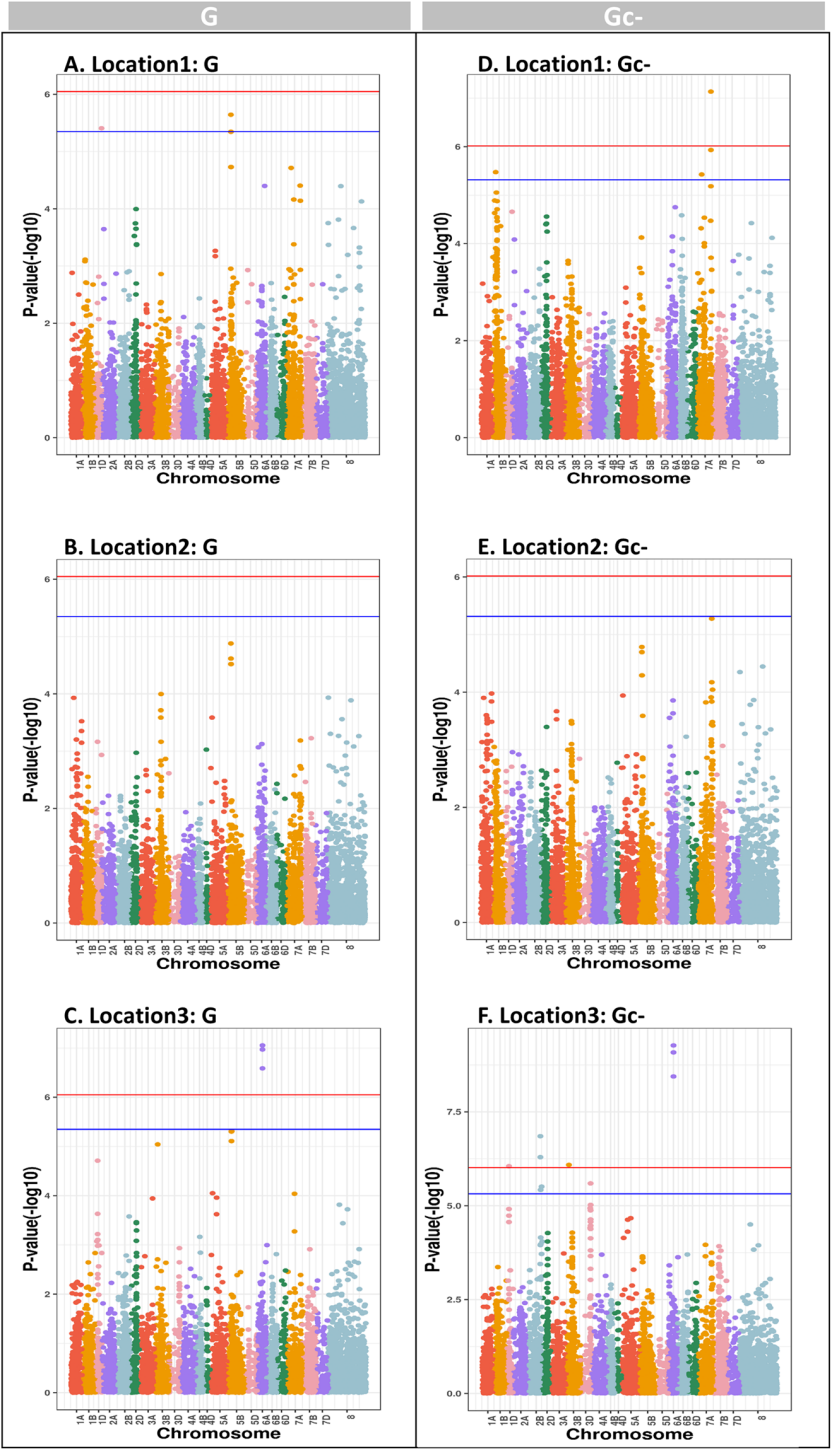
The comparison between three locations in grain yield performance in wheat breeding lines using the GWAS including genotype-location information in the model (See model 2). Location 1 is Skive, location 2 is Dyngby, and location 3 is Holeby in Denmark. The ${\bf{G}}$ is genomic relationship matrix proposed by the first method in VanRaden (2008)^25^, and ${\bf{G}}{}_{{\boldsymbol{c}}-}$ is adjusted genomic relationship matrix using selected markers based on each chromosome^33^. The blue line is the significance threshold at 5%, and the red line is the significance threshold at 1%. Chromosome 8 represents positional unknown SNPs.

To identify SNPs that are significant only at a certain location in yield in winter wheat, the GWAS model including genotype-location information was developed to estimate the SNP effects in three test locations, respectively. Overall, the Manhattan plots showed that the SNP effects were not identical at three locations, respectively (Fig. 6), and the significant SNPs had higher P-value (−log_10_) when the adjusted GRM method (${{\bf{G}}}_{{\boldsymbol{c}}-}$) was fitted in the model (Table 6). Fifteen SNPs reached to genome-wide significance level (P < 0.05) using adjusted GRM method (${{\bf{G}}}_{{\boldsymbol{c}}-}$), in contrast to seven SNPs when using GRM method (${\bf{G}}$)^25^ (Table 6). There were only a few markers commonly found as significance in different locations, for example, one marker (marker name: geSNP7) on chromosome 7A showed high P-value (-log_10_) in both location 1 and location 2. And, two markers on chromosome 5B (marker name: geSNP1 and geSNP3) were QTLs, but these two SNPs only surpassed the genome-wide significance level (P < 0.05) in location 1, not for location 2 and location 3. In general, the GWAS model including genotype-location information showed that some significant markers were location-specific, and usually significantly associated with one location only.

**Table 6:** Genomic-wide significant markers in GWAS including genotype-location information in the model in grain yield performance in winter wheat (P < 0.05). Location 1 is Skive and location 3 is Holeby in Denmark. The ${\bf{G}}$ is genomic relationship matrix proposed by the first method in VanRaden^25^, and ${\bf{G}}{}_{{\boldsymbol{c}}-}$ is adjusted genomic relationship matrix using selected markers based on each chromosome^33^.

Location:Method*	Marker	Chromosome	Position(cM)	P-value
**Location 1:G**	geSNP1	5B	6695.04	2.27E-06
	geSNP2	1D	1306.61	3.92E-06
	geSNP3	5B	6697.51	4.51E-06
**Location 3:G**	geSNP4	6 A	7984.72	8.86E-08
	geSNP5	6 A	7984.72	1.07E-07
	geSNP6	6 A	7984.72	2.60E-07
**Location 1:Gc-**	geSNP7	7 A	9588.77	7.33E-08
	geSNP8	7 A	9588.77	1.17E-06
	geSNP9	1B	625.21	3.35E-06
	geSNP10	7 A	9204.74	3.74E-06
**Location 3:Gc-**	geSNP5	6 A	7984.72	5.37E-10
	geSNP4	6 A	7984.72	8.24E-10
	geSNP6	6 A	7984.72	3.61E-09
	geSNP11	2B	2445.13	1.41E-07
	geSNP12	2B	2445.11	5.08E-07
	geSNP13	3B	3632.52	8.18E-07
	geSNP14	1D	1136.58	8.85E-07
	geSNP15	3D	4533.64	2.54E-06
	geSNP16	2B	2496.88	3.10E-06
	geSNP17	2B	2454.45	3.78E-06

*: No significant markers were identified in Location 2.

### Genomic prediction using rrBLUP and bayesian power lasso model

Genomic prediction was conducted based on the validated SNPs after quality control steps. Prediction accuracies of models were compared using block cross validation with initial crossing year as a block. Each block contained data from two consecutive years (for instance, a block contained data from 2013 to 2014, and so on). For the tested traits in spring barley and winter wheat, the correlation of observed traits and GEBVs, (ρ(**y**_**c**_, **ĝ**)), using two different models (rrBLUP and Bayesian Power Lasso model) are shown in Figs. 2 and 3 for spring barley and winter wheat, respectively.

First of all, the correlation between observed traits and GEBVs evaluated by Bayesian Power Lasso model was higher than rrBLUP model by 13% in ramularia, by 26% in yield, and by 189% in powdery mildew in spring barley (Fig. 2). Secondly, the correlation by using rrBLUP and Bayesian Power Lasso model was similar in winter wheat. The improvement of accuracy of prediction in observed phenotypes were less than 5% when moving from rrBLUP to Bayesian Power Lasso in yield, protein content and Zeleny value (Fig. 3).

## Discussion

In this study, advanced commercial spring barley and winter wheat breeding lines were phenotyped for several traits of interest, and the genotypes were characterized using SNP microarrays. The main results showed that, firstly, in the GWAS, the number of significant SNPs did not increase by using multivariate GWAS method, and we observed common significant SNPs across different traits by multivariate GWAS method. This may enable us to detect the pleiotropic genetic variants for correlated traits. Thirdly, compared with the univariate GWAS results using yield data from all locations in winter wheat breeding program (Fig. 5B, Yield), the GWAS model including genotype-location information identified several genome-wide significant SNPs associated with grain yield in a specific location, (Fig. 6), but very few SNPs that were significant across multiple locations. Secondly, for powdery mildew and grain yield in spring barley, the prediction accuracy of GEBVs in the genomic prediction can be considerably improved by using the Bayesian Power Lasso model compared with using rrBLUP model.

### GWAS to identify SNPs associated with traits in spring barley and winter wheat

#### Spring barley

For spring barley, powdery mildew caused by *Blumeria graminis* f. sp. *h*ordei, is a critical fungal disease in Nordic countries. There are three powdery mildew resistance alleles, including *mlo*-11, *mlo*-9, and *mlo*-5, which have been successfully characterized^28,29^. Our GWAS results showed that there was a major QTL found in chromosome 4H associated with powdery mildew resistance. A similar study reported that a QTL associated with *mlo* alleles was identified on chromosome 4H^30^, which was also verified in the current study (Fig. 4A). There were two markers on chromosome 4H and one marker in an unknown region significantly associated with ramularia leaf spot disease caused by the fungus *Ramularia collo-cygni*, and these three markers were also identified in powdery mildew resistance by using multivariate GWAS (Tables 4 and 5). This QTL may play a pleiotropic role for the powdery mildew and ramularia resistance. For grain yield and quality traits, including yield, lodging, and straw breaking, there was a QTL found in chromosome 4 H, which was different as the QTL associated with powdery mildew resistance. This result indicated that there might be a QTL in LD captured by the genetic markers (Fig. 4D). Additionally, there were several SNPs close to the genome-wide significance threshold among the SNPs with the unknown position, such SNPs may be of interest for further investigation when they can be located in the genome.

#### Winter wheat

For winter wheat, no genome-wide significant markers were identified for protein content, while some markers approaching the genome-wide significance threshold were found located on chromosome 2D, 5B, 6A, and 7B (Fig. 5A). Previous studies have reported markers associated with protein content were mapped on chromosome 2A, 4D, and 7A^11^; 2A, 3A, 4D, 7A, and 7D^31^; and 3B, 5A, and 6A^32^. All above information implied that the protein content is likely controlled by multiple QTLs each with small genetic effects. Besides, due to the Danish agricultural practice, the breeding lines are grown with a low nitrogen fertilizer supplementation, so the protein content of the wheat in this study were low (Table 1). For Zeleny value, no markers were found passing the significance threshold (P < 0.05), but previous studies showed that there were two significant QTLs found on chromosome 1D and 5D^11^. The differences are likely due to different phenotype collection processes. This study applied NIR technique to estimate the Zeleny value by grain analyzer machine (Infratec 1241, FOSS Ltd.), while Kristensen *et al*.^11^ assessed the Zeleny value of each sample based on the standard laboratory procedures (No. ISO 5529, international standard process). Our results showed that different phenotypic recording methods can lead to different SNP effect estimates.

A similar^27^ study reported SNPs associated with starch content mapped on chromosomes 1B, 3B, and a positional unknown region. The markers identified in Cericola *et al*.^27^ was generally similar as we found in this study (except the marker on chromosome 3B). Furthermore, we also identified one genome-wide significant marker on chromosome 5B, and some markers with high P-value(-log_10_) on 6B (Fig. 5B), but they did not surpass the genome-wide significance threshold (P < 0.05).

### GWAS including genotype-location information in the model to evaluate SNP effect in three different locations

For univariate GWAS results in yield in wheat, there were no significant SNPs observed across locations in the across-location association analysis (that is, GWAS based on data from all three locations) (Fig. 5B, Yield). Compared with Fig. 6, where the GWAS model (model 2) was employed to dissect the effect between genotypes and locations for grain yield, there were three significant SNPs identified in location 1, as well as four significant SNPs identified in location 3. Our results thus indicated that the inclusion of genotype by location effects in the GWAS model can also help to map significant SNPs that are linked to QTL with effects at specific location only. This suggested that plant breeders can apply the GWAS model and including genotype-location information in the model to find more genetic variants of interest in different locations.

Two different genomic relationship matries (${\bf{G}}$ and $\,{{\bf{G}}}_{{\boldsymbol{c}}-}$) were used to compare, and identify significant markers in each location, respectively. Previous studies reported that the inclusion of genetic markers in the genomic relationship matrix is likely leading to the loss of statistical power due to double fitting issue. The double fitting issue is that we concurrently included markers in high LD with the markers in question in the genomic relationship matrix^33,34^. Our results showed that the adjusted GRM method $({{\bf{G}}}_{{\boldsymbol{c}}-}$) can increase the SNP detection power, and thus there were more significant SNPs identified in the association analysis (e.g. Fig. 6C,F).

### Genomic prediction using rrBLUP and power lasso model

#### Spring barley

Two models, namely rrBLUP and Bayesian Power Lasso, were applied to predict genomic breeding values, and block cross validation were used to assess the accuracy of predicting phenotype corrected for fixed effects. The correlations (ρ(${\bar{{\bf{y}}}}_{{\bf{c}}}$, **ĝ**)) were approximately 0.2 to 0.5 in spring barley, and 0.2 to 0.3 in winter wheat (Figs. 2 and 3), which were generally moderate correlation. Compared with rrBLUP, due to the different prior assumptions, the Bayesian model is more capable to capture the genetic variances when certain markers have large effects, while most of the markers only have small effects. The accuracy of genomic prediction of yield and powdery mildew showed that the correlations estimated by Bayesian Power Lasso model were higher than rrBLUP (Fig. 2), indicating there might be QTL with large effects on chromosome 4H that were captured by Bayesian Power Lasso model. Compared with the GWAS results, there was a QTL with a large effect identified on chromosome 4H associated with yield and powdery mildew (Fig. 4A,B). Therefore, the prediction accuracy of genomic prediction was improved using Bayesian Power Lasso model, in comparison with rrBLUP.

#### Winter wheat

For the genomic prediction in winter wheat (Fig. 3), the accuracies of predicting GEBVs were generally similar in rrBLUP and Bayesian Power Lasso. Compared with previous studies, similar results were observed in protein content and Zeleny value (*cf*. Table 6 in Kristensen *et al*.^11^). Results indicated that the traits were governed by a complex genetic architecture explained by numerous QTLs with small genetic effects, without any major QTLs existing in the testing population.

## Conclusion

Overall, this study applied GWAS to identify QTL, and used genomic prediction for several traits in commercial spring barley and winter wheat breeding programs. The GWAS analysis successfully identified several markers significantly associated with grain yield, quality traits and disease-resistance traits in spring barley and winter wheat. Furthermore, a clear QTL was found on chromosome 4H for powdery mildew in spring barley. The GWAS including genotype-location information in the model based on different experimental locations indicated that the significant markers associated with the QTLs were different in three experimental locations in the wheat breeding program. In addition, compared with GRM method (${\bf{G}}$), there were more SNPs surpassed the genome-wide significance threshold when using adjusted GRM (${{\bf{G}}}_{{\boldsymbol{c}}-})$ in the GWAS analysis. For genomic prediction, the model comparison showed the Bayesian Power Lasso model outperformed rrBLUP, in terms of accuracy of GEBVs prediction for powdery mildew and yield trait in spring barley. Based on this study we concluded that, first of all, for GWAS analysis, the GWAS including genotype-location information in the model can help to identify QTL that only have effects at specific locations. Secondly, for traits influenced by major QTLs, using Bayesian method is better than rrBLUP for prediction of breeding values.

## Materials and Methods

### Field experiment and phenotype collections

In total, there were 1,317 advanced breeding lines in spring barley, and 1,325 advanced breeding lines in winter wheat involved in this study. The breeding lines of spring barley (*H. vulgare L*.) and winter wheat (*T. aestivum L*.) were phenotyped from four breeding cycles (set 2013, set 2014, set 2015, and set 2016) produced by seed breeding company (Nordic Seed A/S, Galten, DK). Every breeding cycle contained approximately 330 breeding lines for each crop (spring barley and winter wheat), and the F_6_ generation of every line were tested in two consecutive years at three experimental sites (Dyngby, Holeby, and Skive (for the first year only)) in Denmark. A new set of crosses were produced in every year. There were multiple trials nested within each experimental site, and the trial design was randomized complete block design^35^.

For three experimental locations, the climate and soil type are slightly different. The soil types of Dyngby and Skive in Jutland region are sandy loam, and Holeby in Sealand region is loam soil which contains more clay. The yearly average temperature of Jutland region was approximately 0.5 celsius degree lower than Sealand region. The yearly average rainfall were similar, which were approximately 750 mm, in both Jutland and Sealand regions (http://www.dmi.dk/vejr/arkiver/vejrarkiv/). More information in terms of soil texture profiles and field practice can be found in Supplementary Information.

The number of breeding lines of the two crops tested in each trial was slightly different. For spring barley, every trial had 22 breeding lines and three checks with three replicates in the first year, but two replicates in the second year. For winter wheat, every trial contained 21 breeding lines and four checks with two replicates in both years.

The following phenotypes were analyzed:

1. Yield: measured as kg grain / 8.25 m^2^ plot in both crops.
2. Lodging: the trait in spring barley was phenotyped by visual evaluation on a 1 to 9 scale with 1 indicating erect plants, and 9 indicating plants bending completely to the ground.
3. Straw breaking: the trait in spring barley was assessed by visual evaluation on a scale from 1 to 9, with 1 indicating no broken straws, and 9 indicating broken straws for all plants.
4. Powdery mildew and Ramularia: disease traits were recorded in spring barley scored by visual evaluation ranging from 1 to 9, where 1 means less than 0.01% coverage of infection on the leaves, and 9 means more than 75% coverage of infection.
5. Protein content, Starch content, Moisture, and Zeleny sedimentation value: quality traits for protein content and starch content in winter wheat were measured by an instrument based on NIT (Near Infrared Transmission) technology (Grain Analyser for grain and flour: Infratec 1241 (FOSS Ltd.)) (https://www.fossanalytics.com/en#) after harvest. The moisture was to measure the water content (%) of grain after harvest evaluated by NIT as well. The instrument used operates in the near-visible range of 850–1,050 nm, using a monochromator. Last-squares regression was used to compute calibration and composition parameter (Williams, 1991^36^). Zeleny value estimates the degree of sedimentation of flour suspended in a lactic acid solution during a standard time interval and is associated with baking quality. In this study, Zeleny value was measured by NIR (Near Infrared Reflectance) (Perten Instruments, Sweden) (https://www.perten.com/).

### Genomic data and genotyping

Genomic DNA was extracted from leaves of three bulked, two weeks old seedlings for each breeding line using the CTAB approach^37^. The genotypes of DNA samples were subsequently characterized by Illumina 9 K barley iSelect HD SNP-chip for spring barley, and 15 K wheat iSelect HD SNP-chip for winter wheat. There were 4,056 genetic markers in spring barley and 11,154 genetic markers in winter wheat remained for analyses after the following quality control steps: (i) the minor allele frequency < 0.01 and (ii) missing SNP data per line > 0.02. For both crops, the reference genome assembly was used to anchor the markers to current existing linkage groups, accounting for 2,841 SNPs in spring barley and 9,290 SNPs in winter wheat were successfully mapped. There were 1,215 SNPs in spring barley and 1,864 SNPs in winter wheat where their positions were unknown^2,4^.

### Genomic relationship matrix construction

The genomic relationship matrix (GRM) was constructed based on the first method proposed by VanRaden^25^, **G** = **ZZ’/2∑p**_**j**_ **(1-p**_**j**_**)**, where the matrix **Z** was calculated as (**M**−**P**). **M** is a matrix of minor allele counts (0,1,2) with *m* columns (one for each marker) and *n* rows (one for each line). **P** is a matrix which contains the allele frequency, with column *j* defined as ${\bf{l}}2({p}_{j}-0.5)$, where **l** is a vector of ones, and ${P}_{j}$ is the frequency of the minor allele at corresponding locus *j*. The default mean imputation approach was applied to impute the missing value in the genotype data, where there were approximately 1% missing genotyping data existing in both spring barley and winter wheat after quality control steps. The mean imputation approach was applied to impute the missing genotype. The principal component analysis (PCA) applied the genomic relationship matrix (GRM) to the analysis was performed to characterize the genetic relationship between breeding lines in both species.

### Heritability estimation and genomic effect of SNP

For rrBLUP and Bayesian Power Lasso models, the plot heritability was estimated by dividing the additive genetic variance (total variance of GEBVs, ${\sigma }_{a}^{2}$) with total phenotypic variance (${\sigma }_{p}^{2}$). For the genomic effect of SNPs, the percentage of additive genetic variance explained by the SNP was calculated by 2*pq*(**α**)^2^, and divided by the total additive genetic variances, where the *p* and *q* are the major and minor allele frequency of the SNP, and **α** is the substitution effect of SNP based on the single marker regression analysis^38^.

### Cross-validation and prediction accuracy

A block cross validation strategy using crossing set as block was employed to evaluate the accuracy genomic prediction in spring barley and winter wheat breeding programs. The approach was to mask one set (as one block) from all available sets (in this study, we totally have four sets, each set contained data from two consecutive years, e.g. 2013 to 2014 as 1^st^ set.), and to use remaining sets for model parameters estimation, and then predict the breeding value of the masked set. The whole procedure was finished repeatedly until all sets were predicted. The purpose of this strategy is that breeders can apply the same concept to predict the performance of the trait of interest in a future year based on current phenotypic collections. The line correlation (ρ(${\bar{{\bf{y}}}}_{{\bf{c}}}$, **ĝ**)) of the tests were estimated as the correlation between the mean of observed phenotypes corrected for the fixed effects based on the same breeding line (${\bar{{\bf{y}}}}_{{\bf{c}}}$) and GEBVs (**ĝ**). The plot correlation (ρ(**y**_**c**_, **ĝ**)) was to estimate the observed phenotypes corrected for the fixed effects of each plot and GEBVs.

### GWAS

Single marker linear regression models were implemented to identify the association between SNPs and QTLs. The analyses were performed by using the DMU package^39^. The following single SNP regression model was applied:

1 $${\bf{y}}={\bf{X}}{\bf{b}}+{{\boldsymbol{m}}}_{{\boldsymbol{i}}}{B}_{i}+{{\boldsymbol{Z}}}_{1}\,{\boldsymbol{g}}+{{\boldsymbol{Z}}}_{2}\,{{\boldsymbol{f}}}_{1}+{{\boldsymbol{Z}}}_{3}\,{{\boldsymbol{f}}}_{2}+\mathop{\sum }\limits_{i=1}^{9}{{\boldsymbol{Z}}}_{{\boldsymbol{i}}}{\boldsymbol{s}}+{\boldsymbol{e}}$$

where ${\bf{y}}$ is the vector of trait; ${\bf{X}}$ is a design matrix of the fixed factor; ${\bf{b}}$ is a vector of fixed factor (year * location * trial); ${{\boldsymbol{m}}}_{{\boldsymbol{i}}}$ is the vector of genotypes of the ith marker coded as 0, 1, and 2, ${B}_{i}$ is the additive genetic effect of the ith marker; ${{\boldsymbol{Z}}}_{{\boldsymbol{n}}}\,$are the design matrices of random factors; ${\bf{g}}$ is a vector of genomic breeding value for the lines with **g** ~ N(0, **G** ${\sigma }_{g}^{2}$) with ${\sigma }_{g}^{2}$ represents the genomic variance and **G** is the genomic relationship matrix; ${{\boldsymbol{f}}}_{1}$ is a vector of random G × E interaction effects, year * location * line; ${{\boldsymbol{f}}}_{2}$ is a vector of random factor, location * line, both with ${f}_{n}$ ~ N(0, **I** ${\sigma }_{fn}^{2}$); **s** is a vector of spatial effect with **s** ~ N(0, **I** ${\sigma }_{s}^{2}$), which contained eight surrounding plots and plot itself (n=9); **e** is a vector of random residuals with **e** ~ N(0, **I** ${\sigma }_{e}^{2}$).

For yield, lodging, and straw breaking in spring barley, as well as yield, moisture, starch content, protein content, and Zeleny value in winter wheat, were analyzed using model (1). However, for powdery mildew and Ramularia in spring barley, the random factors, location * line (${{\boldsymbol{f}}}_{2}$) and spatial effect (${\boldsymbol{s}}$), were removed from the model due to convergence issues because the corresponding variance components converged towards zero.

The multivariate GWAS were performed using the R package, TATES, with default settings^16^. The original concept was proposed by Li *et al*.^40^ and the procedure was improved by van der Sluis *et al*.^16^. This method was to combine the P-value of the marker derived from univariate GWAS analysis to obtain the one-trait-based P-value, and correcting for phenotypic correlations between all traits involved in the multivariate GWAS analysis. The estimation is computed by, ${P}_{T}=Minium(\frac{{m}_{e}{P}_{j}}{{m}_{ej}})$, where $\,{P}_{T}$ is the smallest weighted P-value with the null hypothesis that none of the traits is associated with the marker, and the alternative hypothesis is that at least one of the traits is associated with the marker; and ${P}_{j}$ is the ${j}^{th}$ sorted P-value of the marker in the ascending order in the matrix; ${m}_{e}$ is the effective numbers of independent P-values of all $m$ phenotypes for a marker; ${m}_{ej}$ is the effective number of P-values among the top $j\,$P-values, where $\,j$ runs from 1 to $m$ phenotype, as described in Li *et al*.^40^ and van der Sluis *et al*.^16^.

Significance tests of marker effects were performed using a two-sided t-test with a null hypothesis of a marker (${B}_{i}$) effect is zero. A Bonferroni correction was used to control the false positive associations in a multiple comparison procedure. The significance level was defined as P-value < 0.05/N and 0.01/N, where 0.05 and 0.01 is the desired overall significance level, and N is the number of testing markers in the analysis^41^.

#### GWAS including genotype-location information in the model

For each unique breeding line in winter wheat, there were multiple plots tested in three different locations (Skive, Dyngby, and Holeby) in Denmark. In order to understand the effect between genotype and yield traits performance in different locations, the GWAS model including genotype-location information was used to estimate SNP effects in each location.

The linear regression model is:

2 $${\bf{y}}={\bf{X}}{\bf{b}}+{{\boldsymbol{m}}}_{{\boldsymbol{i}}}{B}_{i}+{{\boldsymbol{Z}}}_{1}{\boldsymbol{g}}+{\boldsymbol{L}}{{\boldsymbol{g}}}_{{\boldsymbol{L}}}+{\boldsymbol{e}}$$

where ${\bf{y}}$ is the vector of trait; ${\bf{X}}$ is a design matrix of the fixed factor; ${\bf{b}}$ is a vector of fixed factor (year * location * trial); ${{\boldsymbol{m}}}_{{\boldsymbol{i}}}$ is the vector of genotypes of the ith marker coded as 1, 0, and −1, ${B}_{i}$ is the additive genetic effect of the ith marker; ${{\boldsymbol{Z}}}_{1}$is the design matrix of random factor; ${\bf{L}}$is the design matrix for each location, respectively; ${\bf{g}}$ is a vector of genomic breeding value for the lines with **g** ~ N(0, **G** ${\sigma }_{g}^{2}$) with ${\sigma }_{g}^{2}$ represents the genomic variance, and **G** is the genomic relationship matrix; ${{\boldsymbol{g}}}_{{\boldsymbol{L}}}\,$is a vector of genomic breeding value for a line in each location (${\boldsymbol{L}}$) with **g**_**L**_ ~ N(0, **G** ${\sigma }_{gL}^{2}$); and **e** is a vector of random residuals with **e** ~ N(0, **I** ${\sigma }_{e}^{2}$).

Except for the genomic relationship matrix (${\bf{G}}$) which is constructed using VanRaden^25^ first method, the adjusted genomic relationship matrix (${{\bf{G}}}_{{\boldsymbol{c}}-})$ was constructed depending on the chromosome position of the marker, to correct the double fitting issue between the testing marker and overall genomic information in the model. For example, when we performed the GWA analysis for those markers mapped on chromosome 1, the adjusted genomic relationship matrix (${{\bf{G}}}_{{\boldsymbol{c}}-}$) was made by those markers that were mapped on the rest of chromosomes except chromosome 1, and so on for rest of SNPs on other chromsomes^33^.

### Genomic prediction

The genomic prediction was performed using the Bayesian Power Lasso model and ridge regression BLUP (rrBLUP) model. The Bayesian Power Lasso model is:

3 $${\bf{y}}={\bf{X}}{\bf{b}}+{{\boldsymbol{Z}}}_{1}{\boldsymbol{u}}+{{\boldsymbol{Z}}}_{2}{\boldsymbol{l}}+{{\boldsymbol{Z}}}_{3}\,{{\boldsymbol{f}}}_{1}+\mathop{\sum }\limits_{i=1}^{9}{{\boldsymbol{Z}}}_{{\boldsymbol{i}}}{\boldsymbol{s}}+{\boldsymbol{e}}$$

where ${\bf{y}}$ is a vector of observed phenotypes, ${\bf{b}}$ is a vector of the mean plus year * location * trial effect with design matrix ${\bf{X}}$ assuming as uniform distribution; ${\boldsymbol{u}}$ is a vector of additive genetic effects and ${{\boldsymbol{Z}}}_{1}$ is a matrix of the alleles of the SNPs coded as 0, 1, and 2; ${{\boldsymbol{l}}}_{}$ is a vector of random factor of line effects with design matrix ${{\boldsymbol{Z}}}_{2}$; G × E effect $({{\boldsymbol{f}}}_{1}$), and spatial effect **(s)** are the same as described in model (1); **e** is a vector of random residuals. Random residuals were assigned a normal prior distribution. The variances and rate parameter, ${\lambda }_{RP}$, were assigned flat prior distributions. The prior distribution of SNP effects was assigned to be an exponential power distribution as below^42^:

4 $$p({\boldsymbol{u}})=\mathop{\prod }\limits_{i=1}^{m}\frac{1}{2}{\lambda }_{RP}{e}^{-{\lambda }_{RP}{|{u}_{i}|}^{\beta }}$$

where $m$ is a number of markers, and ${\rm{\beta }}$ is shape parameter to control the sparsity, affecting the shrinkage of the SNP effects. When ${\rm{\beta }}$ is set to 1, the analysis is identical to standard Bayesian Lasso, and the absolute SNP effect, $|{u}_{i}|$, are assumed to be exponential distribution. When ${\rm{\beta }}$ is less than 1, the larger difference between SNPs can be seen when a relatively sharper and longer-tailed distribution is used, following by higher sparsity in the SNP effects, and intense shrinkage in contrast to standard Bayesian Lasso (${\rm{\beta }}$ = 1). The shape parameters were set to be 0.4, 0.8 or 1.0 in the analyses at the initial test, to select the most suitable shape parameter for analysis. Deviance Information Criterion was computed for every test, and further to optimize the ${\rm{\beta }}$ for each trait^43^. The Bayesian analyses were performed as the single chain, with a total length of 1,000,000 rounds of the Markov chain Gibbs sampling, and the first 200,000 cycles discarded as burn-in. Every 40^th^ sample of the rest of the cycles were kept for posterior analysis (skip interval = 40). The Bayesian model analyses were performed using Bayz software^44^. Posterior means and convergence were computed using the scripts supplied with Bayz and R package CODA^45^.

The rrBLUP model used in this study is:

5 $${\bf{y}}={\bf{X}}{\bf{b}}+{\boldsymbol{Wa}}+{{\boldsymbol{Z}}}_{1}{\boldsymbol{l}}+{{\boldsymbol{Z}}}_{2}\,{{\boldsymbol{f}}}_{1}+\mathop{\sum }\limits_{i=1}^{9}{{\boldsymbol{Z}}}_{{\boldsymbol{i}}}{\boldsymbol{s}}+{\boldsymbol{e}}$$

where ${\bf{y}}$ is a vector of observed phenotypes, ${\bf{b}}$ is a vector of the mean plus year*location*trial effect with design matrix ${\bf{X}}$; ${\boldsymbol{W}}$ is a design matrix, allocating records to genotypes for all testing markers; ${\boldsymbol{a}}$is the vector of regression coefficients for random SNP effects; ${{\boldsymbol{l}}}_{}$ is a vector of random factor of line effects with design matrix ${{\boldsymbol{Z}}}_{1}$; G × E effect $({{\boldsymbol{f}}}_{1}$), and spatial effect **(s)** are the same as described in model (1); **e** is a vector of random residuals with N(0, **I** ${\sigma }_{e}^{2}$).

## Supplementary information

Supplementary Information.
