# Author Correction: Genomic prediction and GWAS of yield, quality and disease-related traits in spring barley and winter wheat

**DOI:** 10.1038/s41598-020-63862-3

**Published:** 2020-05-13

**Authors:** Hsin-Yuan Tsai, Luc L. Janss, Jeppe R. Andersen, Jihad Orabi, Jens D. Jensen, Ahmed Jahoor, Just Jensen

**Affiliations:** 1 Department of Marine Biotechnology and Resources, National Sun Yat-Sen University, Kaohsiung City, Taiwan; 2 Center for Quantitative Genetics and Genomics, Department of Molecular Biology and Genetics, Aarhus University, Tjele, Denmark; 3 Nordic Seed, Galten, Denmark; 4 Department of Plant Breeding, Swedish University of Agricultural Sciences, Alnarp, Sweden

Correction to: *Scientific Reports* 10.1038/s41598-020-60203-2, published online 25 February 2020

This Article contains errors in Tables 4 and 5, where in the “Trait” column in Table 4 and the “Trait Combination” column in Table 5, the row title “Straw breaking” for the barley species was incorrectly given as “Starch”.

The correct Tables 4 and 5 appear below.

**Table 4:** Genome-wide significant markers in univariate GWAS in spring barley and winter wheat (P < 0.05).

	Trait	Marker	Chromosome	Position(cM)	P-value	Genomic Effect*
Barley	Powdery Mildew	SNP1	4H	100.68	1.09E-20	10.70
		SNP2	4H	101.28	1.72E-19	10.30
		SNP3	4H	99.68	2.73E-19	10.00
		SNP4	4H	102.38	9.75E-19	10.20
		SNP5	4H	101.68	1.23E-13	6.70
		SNP6	4H	102.08	1.68E-11	5.55
		SNP7	4H	102.18	1.95E-11	5.53
		SNP8	Unknown	Unknown	3.89E-10	5.03
	Ramularia	SNP6	4H	102.08	2.69E-07	7.03
		SNP7 SNP9	4H Unknown	102.18 Unknown	2.92E-07 4.01E-07	7.00 6.11
	Yield	SNP10	4H	88.84	5.85E-07	0.75
		SNP11	4H	88.84	5.85E-07	0.75
		SNP12	4H	88.84	5.85E-07	0.75
		SNP13	4H	88.64	1.76E-06	0.68
	Straw breaking	SNP14	4H	32.24	6.19E-08	18.60
	Lodging	SNP14	4H	32.24	4.44E-09	12.70
Wheat	Moisture	SNP15	6A	40.8	1.22E-06	1.34
		SNP16	1B	201.25	2.16E-06	1.44
		SNP17	6A	30.04	3.13E-06	1.23
	Starch Content	SNP18	5B	222.57	4.17E-06	4.09

*The genomic effect value is multiplied by 10^3^.

**Table 5:** Genome-wide significant markers in multivariate GWAS in spring barley and winter wheat (P < 0.05).

	Trait Combination	Marker	Chromosome	Position(cM)	P-value
Barley	Mildew Ramularia	SNP1	4H	100.68	2.18E-20
		SNP2	4H	101.28	3.44E-19
		SNP3	4H	99.68	5.46E-19
		SNP4	4H	102.38	1.95E-18
		SNP5	4H	101.68	2.45E-13
		SNP6	4H	102.08	3.36E-11
		SNP7	4H	102.18	3.91E-11
		SNP8	Unknown	Unknown	7.78E-10
		SNP19	4H	97.38	2.59E-08
		SNP20	4H	98.65	6.51E-07
		SNP9	Unknown	Unknown	8.01E-07
		SNP21	4H	97.29	6.64E-06
		SNP22	4H	97.19	6.70E-06
		SNP23	4H	97.19	9.18E-06
	Yield	SNP14	4H	32.24	1.27E-08
		SNP10	4H	88.84	1.68E-06
	Straw breaking	SNP11	4H	88.84	1.68E-06
		SNP12	4H	88.84	1.68E-06
	Lodging	SNP13	4H	88.64	5.04E-06
Wheat	Moisture Starch Yield	SNP15	6A	40.8	3.57E-06

*The markers were not identified in univariate GWAS (cf. Table 4).

