# Supplementary material for: Genomic prediction and GWAS of yield, quality and disease-related traits in spring barley and winter wheat
Source: Sci Rep. 2020 Feb 25;10:3347. doi: 10.1038/s41598-020-60203-2 (PMC7042356; doi:10.1038/s41598-020-60203-2)
Supplement: Supplementary file 1 — Supplementary Information. [file 41598_2020_60203_MOESM1_ESM.pdf]

Supplementary Information

Genomic prediction and GWAS of yield, quality and disease-related traits in spring barley and winter wheat

Hsin-Yuan Tsai[1,2*], Luc L. Janss[2], Jeppe R. Andersen[3], Jihad Orabi[3], Jens D. Jensen[3], Ahmed Jahoor[3,4], Just Jensen[2]

Affiliations

[1]Department of Marine Biotechnology and Resources, National Sun Yat-Sen University, Kaohsiung City, Taiwan

[2]Center for Quantitative Genetics and Genomics, Department of Molecular Biology and Genetics, Aarhus University, Tjele, Denmark

[3]Nordic Seed, Galten, Denmark

[4]Department of Plant Breeding, Swedish University of Agricultural Sciences, Alnarp, Sweden

Supplemental Materials 1. Supplemental Materials and Methods

Field practice: field management, irrigation system, weeding and fertilization

Regarding the **field management**, the trial plots were sown in a plowed seed bed with a combined rotor harrow seeder implement. Seed percentage was 300 germinating seed pr. m^2 sown in a $10m^2$ plot. At harvest, the size of plot was about $8.25m^2$. **Irrigation**: No irrigation was used. **Weeding**: Weed control was carried out with herbicides, according to the weeds presenting at the given locations. **Fertilizer**: The fertilizer usage was according to Danish Regulation of nutrients in Agriculture (Environmental Protection Agency, Ministry of Environment and Food of Denmark, 2017). Fertilization was done with NPK 21-3-10 + 1% Mg, and 4% S fertilizer, 600 kg per hectare which contains 126 kg N/ha, 18 kg P/ha, 60 kg K/ha, 1 kg Mg/ha and 24 kg S/ha. All plots were received two times fungicide treatment (for spring barley), 0.25 l/ha Prosaro (including Prothioconazole 125g/l and Tebuconazole 125 g/l), and 0.25 l/ha Comet pro (Pyraclostrobin 200 g/l) at the stage of BBCH 31-33 and BBCH 39-45.

Soil texture profile of three experimental locations (Dyngby, Skive, and Lolland)

According to Danish agricultural soil type classification (Madsen et al., 1992), Dyngby is JB6 type (10-15% clay, 0-30% silt, 40-90% fine sand, 55-90% coarse sand, and 0-10% humus), Skive is JB4 type (5-10% clay, 0-25% silt, 40-95% fine sand, 65-90% coarse sand, and 0-10% humus) and Lolland is JB7 type (15-25% clay, 0-35% silt, 40-85% coarse sand, and 0-10% humus).

Grain harvest method

The grain were harvested by using a plot harvest combiner with a weighing unit for quantification of plot yield in kilogram. The sample was kept in a closed container for moisture content determination.

Software section

For the software used in this study, the linear regression model (including rrBLUP, single trait GWAS, and GWAS including genotype-location information in the model) was performed using DMU package (Madsen et al., 2013), which was maintained by our institute (DMU package can be downloaded from https://omictools.com/dmu-tool and free for research use only). The multivariate GWAS were

performed using the R package, TATES, with default settings (van derSluis et al., 2013) (TATES software is available from http://ctglab.nl/software). The original concept was proposed by Li et al. (2011), and the procedure was improved by van der Sluis et al. (2013). The Bayesian model analyses were performed using Bayz software (Bayz software is available from http://www.bayz.biz/ managed by Luc Janss in our institute). Posterior means and convergence were computed using the scripts supplied with Bayz and R package named as ‘CODA’ (Plummer et al., 2006).

Reference

Henrik Breuning Madsen, Axel Henry Nørr, and Kristian Aagaard Holst. 1992. Atlas of Denmark Series I, Volume 3 The Danish Soil Classification, 56 p.

Janss, L. L. Bayz Genomic Model Analysis Package. http://www.bayz.biz/ (2019).

Li, M.-X., Gui, H.-S., Kwan, J. S. H. &Sham, P. C. GATES: a rapid and powerful gene-based association test using extended Simes procedure. *Am. J. Hum. Genet.* **88**, 283–293 (2011).

Madsen, P. &Jensen, J. *DMU: A user's guide. A Package for Analysing Multivariate Mixed Models. Version 6. Release 5.2.* (2013).

Plummer, M., Best, N., Cowles, K. &Vines, K. CODA: convergence diagnosis and output analysis for MCMC. *R News* **6**, 7–11 (2006).

Overview of the Danish regulation of nutrients in agriculture and the Danish Nitrates Action Program. June 2017. Environmental Protection Agency, Ministry of Environment and Food of Denmark, Denmark.

van derSluis, S., Posthuma, D. &Dolan, C.V. TATES: Efficient Multivariate Genotype-Phenotype Analysis for Genome-Wide Association Studies. *PLOS Genet.* **9**, e1003235 (2013).

Supplemental Materials 2. Q-Q plot of association analysis.

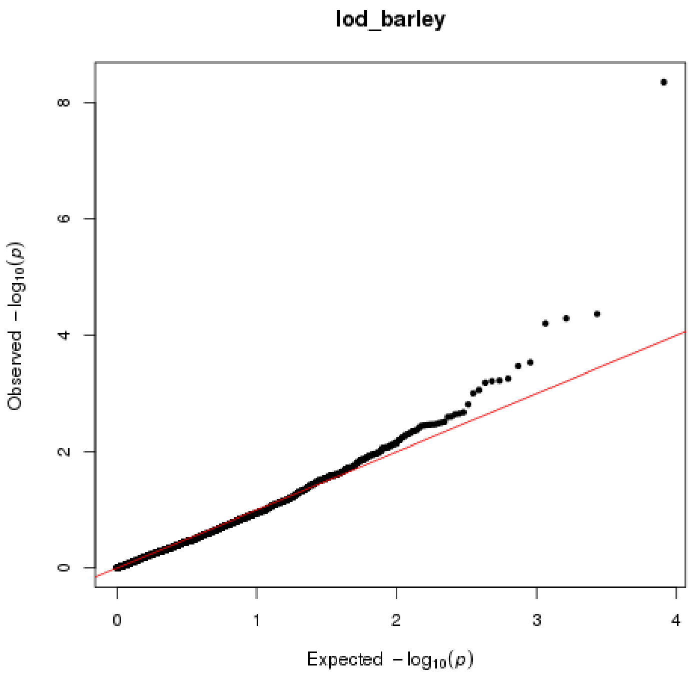

Figure 1. Q-Q plot for lodging in spring barley.

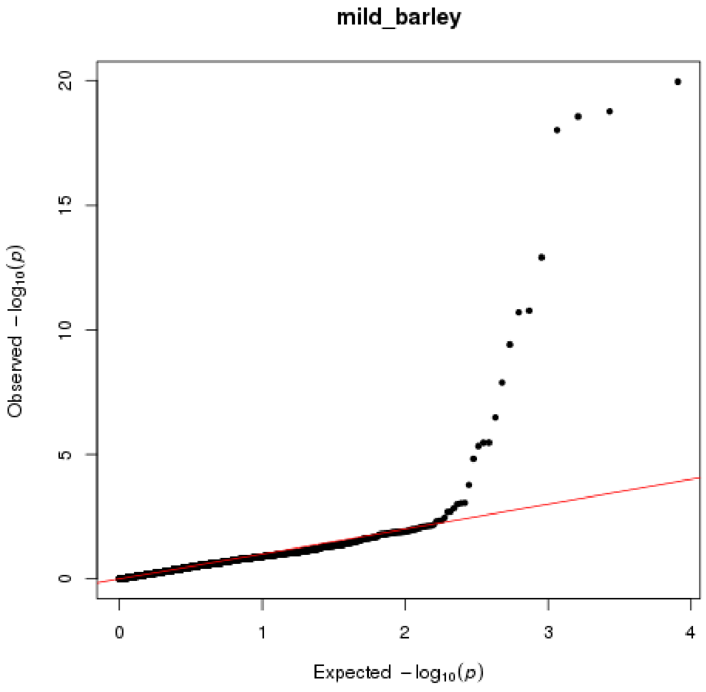

Figure 2. Q-Q plot for powdery mildew in spring barley.

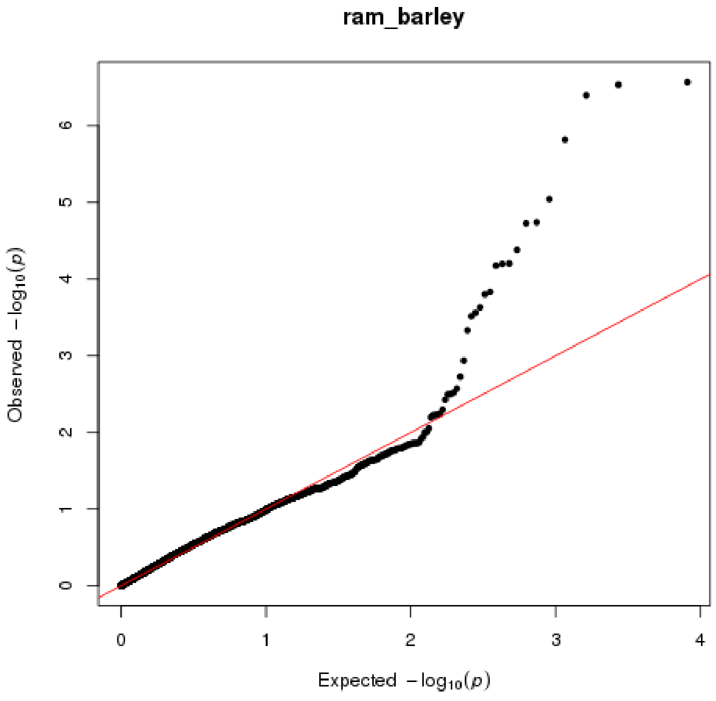

Figure 3. Q-Q plot for ramularia in spring barley.

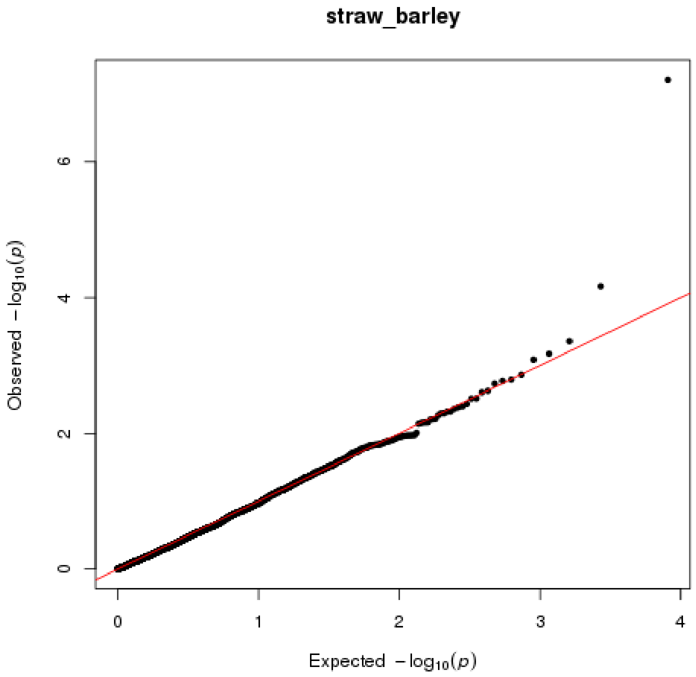

Figure 4. Q-Q plot for straw breaking in spring barley.

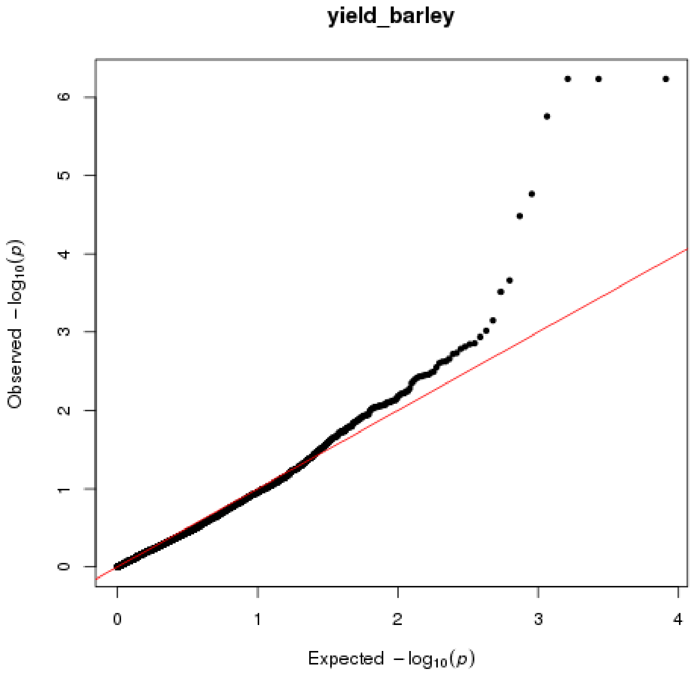

Figure 5. Q-Q plot for yield in spring barley.

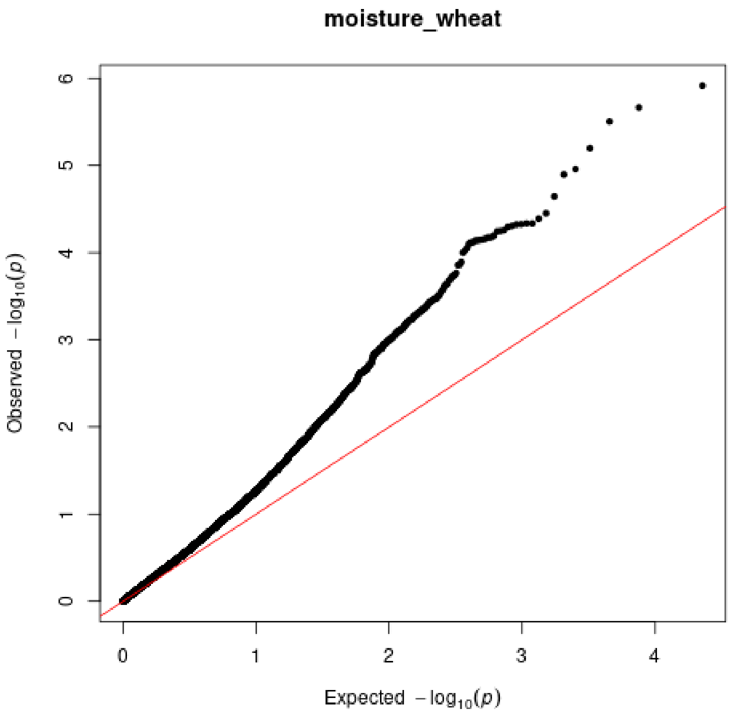

Figure 6. Q-Q plot for moisture in winter wheat.

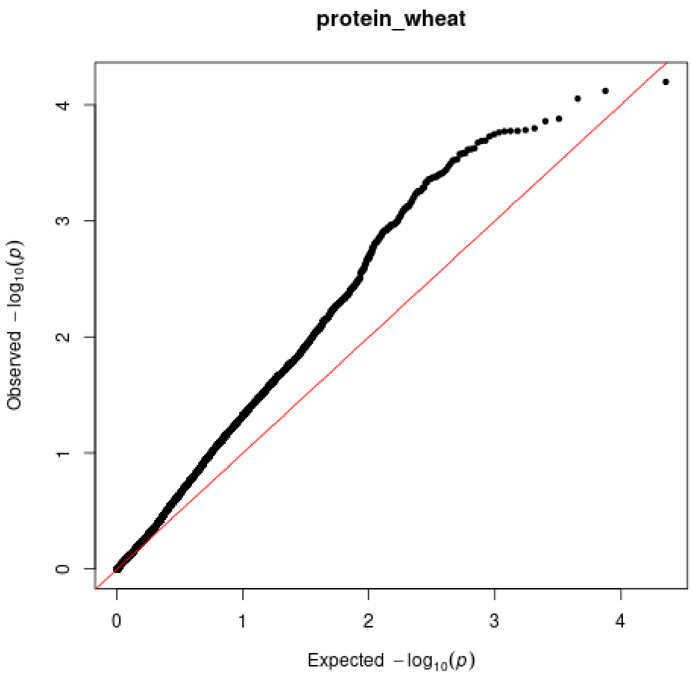

Figure 7. Q-Q plot for protein content in winter wheat.

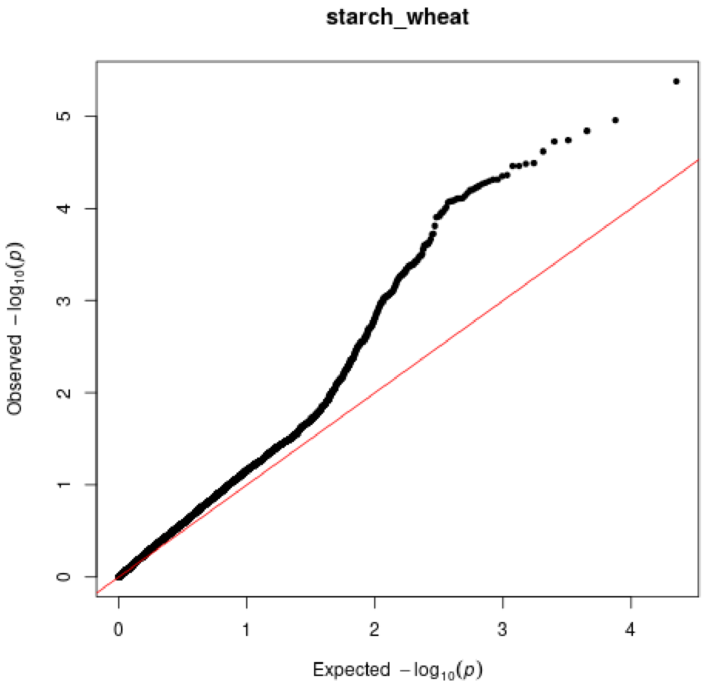

Figure 8. Q-Q plot for starch content in winter wheat.

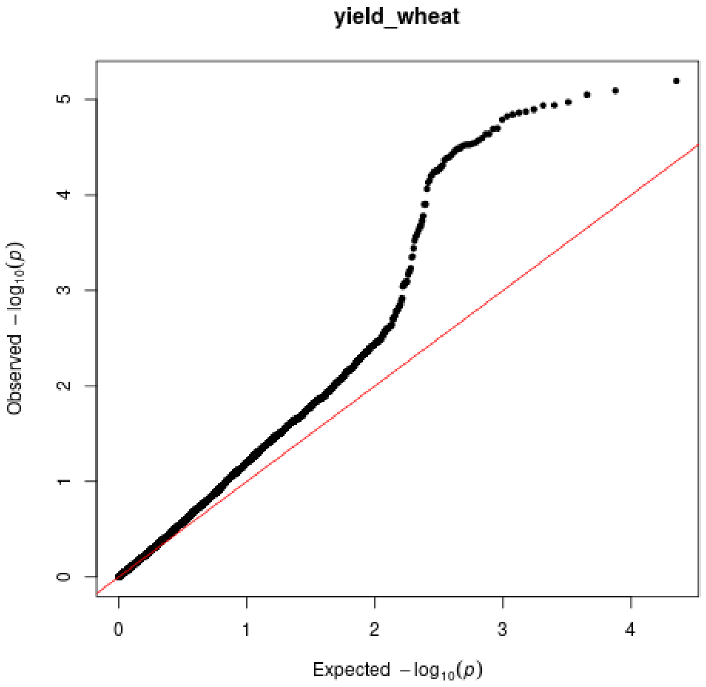

Figure 9. Q-Q plot for yield in winter wheat.

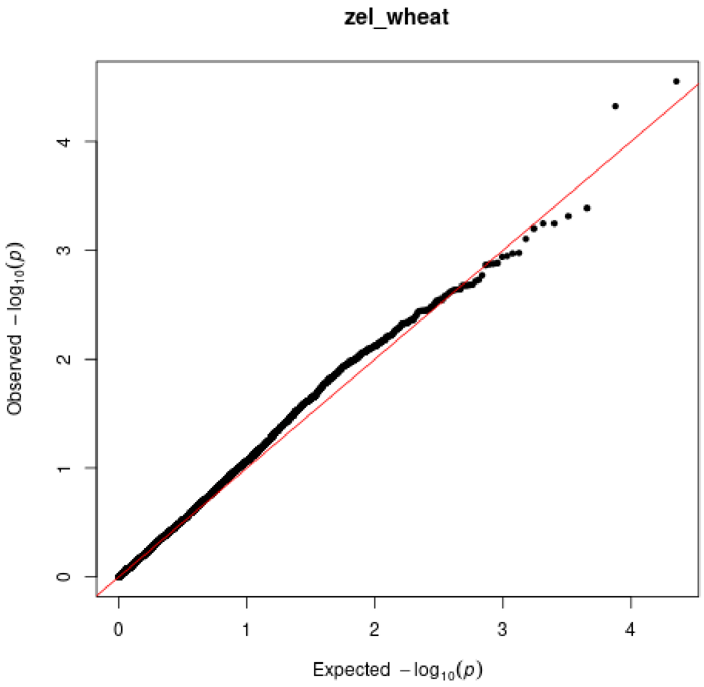

Figure 10. Q-Q plot for Zeleny value in winter wheat.
